# What’s new in cancer and molecular imaging

**DOI:** 10.1016/j.ejro.2022.100437

**Published:** 2022-08-24

**Authors:** Lacey J. McIntosh

**Affiliations:** University of Massachusetts Chan Medical School / University of Massachusetts Memorial Health Care, Division of Oncologic and Molecular Imaging, Department of Radiology, 55 Lake Avenue, North, Worcester, MA 01655, USA

**Figure:**
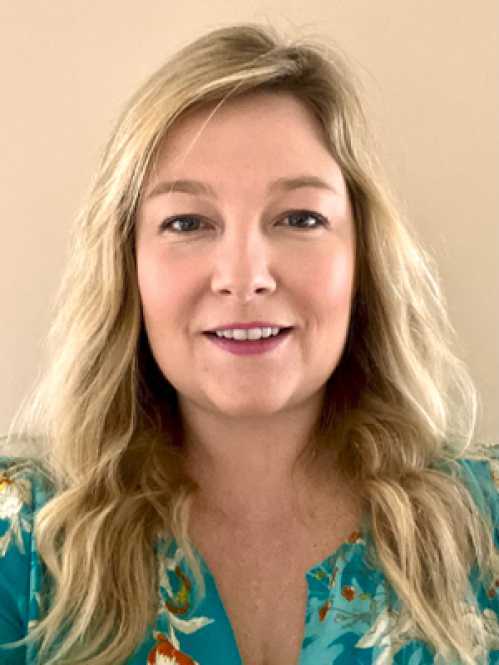

.

Guest Editorial and Introduction to the Special Issue: What’s New in Cancer and Molecular Imaging.

While the imaging of cancer permeates throughout all sections and modalities of radiology, “cancer imaging” is an emerging area of unique training and practice. As the clinical diagnosis and treatment of cancer continues to rapidly evolve, becoming more individualized and precise, analogous changes are occurring in cancer imaging and intervention. Our Special Issue on “What’s New in Cancer and Molecular Imaging” is a collection of articles aiming to serve as an overview of broad topics in cancer imaging and intervention. Our hope is that the reader will have an enhanced understanding of clinical changes in cancer care, which result in new, exciting, and sometimes unexpected changes on imaging, and how this must impact our interpretations and recommendations.

As pathologic assessments and treatment plans become more specific and granular, a parallel evolution is happening in radiology. In addition to focusing on tumor type- and histology-specific features and patterns, we explain how precision oncology practice acknowledges novel types of therapies being used in individualized cancer treatment, the respective toxic manifestations, and response patterns which may be atypical due to unique mechanisms of action. We highlight advancements in screening for hereditary cancers as well as novel molecular targeted imaging, filling previously unmet needs and enhancing diagnostics to a level never seen before. Naturally coupled with the creation of molecular diagnostic tracers is the corresponding development of complementary theranostic agents, allowing for highly specific, precisely delivered, minimally invasive treatments. Articles in this collection discuss the intersection of medical, radiation, and interventional oncology, exploring synergies between local and systemic treatments. New options for local, minimally invasive, and targeted treatments allow more patients more options for treatments than ever before, especially those who may have previously been excluded due to poor candidacy or limited options. In addition to the changes in our diagnostic and clinical practices, we also discuss how to incorporate these concepts into teaching the next generation of radiologists and cancer imagers. Finally, we explore the current and expanding roles of artificial intelligence in cancer imaging.

Imagers are key players on the cancer care team and must keep current with the rapid changes in cancer care; our reports must go beyond descriptions of “better, same, or worse” and consider the entire clinical context to provide high-level, sophisticated, and meaningful interpretations to our clinical colleagues. This area is an opportunity for radiologists to shine and highlight our value on the team. We now need to know more clinical information than ever before, and I speak for all authors in this Special Issue when I say we sincerely hope that you find these articles interesting and informative, and that this content helps elevate your skills in caring for your cancer patients.

A special thank you to my fellow cancer imager and former co-fellow, Dr. Marta Braschi-AmirFarzan, for her invaluable connections in the cancer imaging community, and who made this collection possible.

Sincerely,

Lacey J. McIntosh, DO, MPH.
